# Efficacy of erythrocyte apheresis in the management of severe high-altitude deacclimatization syndrome: a retrospective cohort study

**DOI:** 10.3389/fphys.2025.1622092

**Published:** 2025-09-10

**Authors:** Xinmiao Wang, Rong Huang, Chunrong Su, Hao Yao, Ling Zhang, Hui Yang, Xiao Wang, Yi Su, Hai Yi

**Affiliations:** ^1^ Department of Hematology, The General Hospital of Western Theater Command of Chinese People’s Liberation Army, Chengdu, China; ^2^ Department of Disease Control and Prevention, The General Hospital of Western Theater Command of Chinese People’s Liberation Army, Chengdu, China

**Keywords:** high altitude polycythemia, erythrocyte apheresis, high altitude deacclimatization syndrome, oxygen toxicity, high altitude regions

## Abstract

**Background:**

High-altitude deacclimatization syndrome (HADAS, commonly known as Oxygen toxicity) manifests through multi-organ system dysfunction and significantly deteriorates the quality of life. Unfortunately, there are no effective methods for serious HADAS (SHADAS) patients. To address this urgent public health challenge, we explored the therapeutic potential of erythrocyte apheresis (EA)-a technique previously used in HAPC treatment, and evaluated its efficacy in SHADAS patients.

**Methods:**

This retrospective cohort study analyzed clinical data of 71 SHADAS patients undergoing EA between October 2016 and September 2024 in our hematology department. Complete blood count parameters were serially measured before and after EA. Data analysis was performed using GraphPad Prism 8.02 software.

**Results:**

The study cohort exhibited significant reductions in hemoglobin (HGB) levels after initial EA: HGB decreased from 224.75 ± 12.76 g/L to 172.92 ± 22.60 g/L (males, *P* < 0.001) and from 205.13 ± 12.41 g/L to 164.75 ± 19.26 g/L (females, *P* < 0.001). Notably, 18 patients required repeat EA due to suboptimal response, and these patients had higher HGB baseline levels. (HGB 238.47 ± 8.76 vs 219.67 ± 9.98 g/L). All subjects demonstrated marked symptomatic improvement in SHADAS manifestations including headache, somnolence, and fatigue after EA. Based on these findings, we developed a strategy for the formulation of EA parameter protocols.

**Conclusion:**

Although it remains challenging to prevent SHADAS in individuals transitioning to lower altitude regions, our study demonstrated that EA could rapidly reduce HGB and alleviate associated symptoms. Moreover, we have established the parameters of EA in the treatment of SHADAS.

## Background

High altitude erythrocytosis (HAPC) is characterized by a moderate increase in red blood cell count and blood viscosity within high altitude hypoxic environments, triggering a cascade of pathophysiological changes that impair adaptation to high altitude habitats (Alkhaldy et al., 2024). According to the criteria of the sixth International Conference on High Altitude Medicine and Physiology in 2004, HAPC is defined as the HGB >210 mg/dL in males and >190 mg/dL in females who have lived at the elevation >2500 m for a long time (Milledge, 2004). However, when HAPC patients relocate to lower altitudes or sea level, they may develop varying degrees of high-altitude deacclimatization syndrome (HADAS, commonly known as Oxygen toxicity), manifesting as headache, somnolence, fatigue, dizziness, palpitation, insomnia, chest tightness, memory loss, loss of appetite, general discomfort and other symptoms (Tan et al., 2024; Sun et al., 2023). Although most HADAS cases achieve spontaneous remission, a subset of patients with severe and intolerable symptoms necessitate urgent therapeutic intervention or immediate return to high altitude regions. Without intervention, these symptoms may persist for weeks or even months, with a gradual decrease in red blood cells and HGB in the process (He et al., 2019).

The primary mechanisms underlying HADAS involve a series of metabolic and functional adaptations in the body as it transitions from high altitude hypoxic to low altitude normoxic environments, including reductions in erythrocyte count, hemoglobin levels, and hormonal fluctuations (Deere and Chown, 2006). These adaptations may involve lipid metabolism, inflammatory signaling and coagulation systems (Tan et al., 2024; Paul et al., 2023). Therefore, HAPC patients, particularly those with chronic mountain sickness, exhibit a significantly higher incidence of HADAS compared to the general population, characterized by more severe symptoms, prolonged recovery duration, and more pronounced hematological abnormalities. These findings suggest that HADAS imposes greater physical burdens on HAPC patients. In fact, some HAPC patients with serious HADAS (SHADAS) are forced to return to high altitude regions due to intolerable symptoms. Epidemiological studies highlight substantial heterogeneity in HADAS incidence, attributed to variations in altitude exposure and residence duration. For example, individuals residing at high altitudes for over a decade exhibit a HADAS incidence exceeding 70% (He et al., 2019). Across populations, HADAS incidence generally ranges from 50% to 80% under non-specialized conditions (He et al., 2013). Importantly, approximately 81.6 million people live at elevations above 2,500 m (Tremblay and Ainslie, 2021). This demographic reality translates to a significant absolute number of HADAS cases upon migration to low altitude regions. Consequently, advancing targeted strategies for HADAS management, particularly severe cases (SHADAS), represents a critical public health priority.

To date, substantial research on HAPC has yielded important insights, but the underlying mechanisms of HADAS remain incompletely understood. He *et al.* demonstrated that serum IL-17A levels serve as a novel predictive index for HADAS, and found that Shenqi pollen capsules alleviate HADAS by suppressing reoxygenation injury and inflammatory response (He et al., 2019; He et al., 2016). This conclusion was further validated by a randomized, placebo-controlled trial showing that Shenqi Pollen Capsules improve HADAS symptoms (Shi et al., 2011). Additionally, this study identified two other compound Chinese herbal preparations with inhibitory effects on HADAS and elucidated their specific mechanisms. Mainly, Shenqi pollen capsule principally attenuated dizziness, fatigue, weakness; Rhodiola rosea capsule mainly inhibited symptoms including fatigue, drowsiness, chest tightness; and Sankang capsule primarily alleviated dizziness, fatigue, and palpitations (Shi et al., 2011). Notably, among the three compound Chinese herbal preparations, Shenqi pollen capsule exhibited the highest symptom improvement rate for HADAS. However, even with effective medications, HADAS symptom resolution requires prolonged time, and no current methods exist to rapidly eliminate these symptoms. For HADAS patients, especially SHADAS patients, the process is extremely painful and lengthy.

To develop rapid therapeutic strategies for alleviating HADAS symptoms, we focused on blood cell separation technology-a method capable of directly isolating specific blood components to modulate hematologic parameters (Ma et al., 2024). In this study, we performed erythrocyte apheresis (EA) in SHADAS patients with accompanied HAPC and found that EA rapidly reduced HGB content. Importantly, EA significantly improved SHADAS symptoms within a short timeframe. These results suggest that EA represents a promising therapeutic modality for SHADAS patients with HAPC.

## Materials and methods

### Patient cohort

Patients admitted to the Department of Hematology in The General Hospital of Western Theater Command of Chinese People’s Liberation Army between October 2016 and September 2024 were retrospectively analyzed. All patients met the diagnostic criteria for HAPC, and experienced intolerable symptoms of HADAS after returning to Chengdu Plain (Milledge, 2004; He et al., 2019).

### Procedures

Before EA, all patients underwent a routine physical examination, including complete blood count, coagulation function tests, biochemical indicators, and quantitative scoring of HADAS symptoms. The blood samples were collected using a Fresenius Kabi Ag COM.TEC blood cell separator, and the flow velocity was maintained at 40–50 mL/min, circulation volume 1000–3000mL, and the hematocrit (HCT) parameter was set at 45%. Usually 1000–1600 mL red blood cells were collected, with an expected HGB reduction of 30–50 g/L. ACD was used as the anticoagulant, supplemented with 500–1000 mL of hydroxyethyl starch (volume-matched to the blood removed), 500 mL of normal saline, and 10–20 mL of 10% calcium gluconate (administered as needed). The target HGB level were set at <200 g/L for males and <190 g/L for females. If post-EA HGB levels did not meet the target, repeat EA was performed the following day as indicated. Furthermore, Complete blood count and other indicators were evaluated and compared before and after EA.

### Criterion of curative effect

Quantifiable indicators such as HGB was recorded depend on numerical values. The classification and scoring criteria of the symptoms of HADAS were depend on (He et al., 2019). Each patient’s pre-EA indexs was used as baseline and compared with the post-EA.

### Statistical analysis

GraphPad Prism 8.02 software was used for statistical analysis of the data. According to the type of data, t-test and chi-square test were used. * means P < 0.05, ** means P < 0.01, * and ** means the difference was statistically significant.

## Results

### Patient characteristics

**FIGURE 1:**
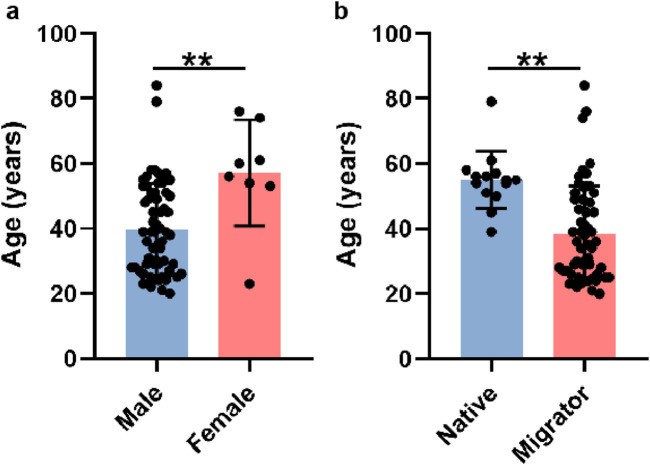
Basic characteristics of participants. **(a)** The average ages of male and female patients. **(b)** The average ages of native residents and migrants.

A total of 71 patients meeting the predefined inclusion/exclusion criteria were enrolled in this study. Demographic analysis revealed a pronounced male predominance (63 males vs Eight females, 88.7% vs 11.3%), which may reflect occupational or sociocultural factors (male-biased migration patterns) rather than a gender-specific predisposition to HADAS. Female participants were significantly older than their male counterparts (mean age: 57.13 ± 16.29 years vs 39.70 ± 14.16 years, P < 0.01; Figure 1a; Table 1). To evaluate potential confounders, patients were further stratified by geographic origin (native high altitude residents vs migrants). Among males, 13 were native to high altitude regions, while 50 were migrants; only one female participant was a native resident. Notably, native patients presented at an older age compared to migrants (54.54 ± 9.07 years vs 35.84 ± 12.64 years, P < 0.0001; Figure 1b), suggesting prolonged high altitude exposure may influence clinical presentation.

**TABLE 1:** Basic characteristics of participants.

Characteristics	Male (n = 63)	Female (n = 8)
Age (years, mean ± SD)	39.70 ± 14.16	57.13 ± 16.29
Naives (n, %)	13 (20.63%)	1 (12.50%)
Migrators (n, %)	50 (79.37%)	7 (87.50%)

Age and region of different gender patients.

### EA improved HAPC -related parameters

**FIGURE 2:**
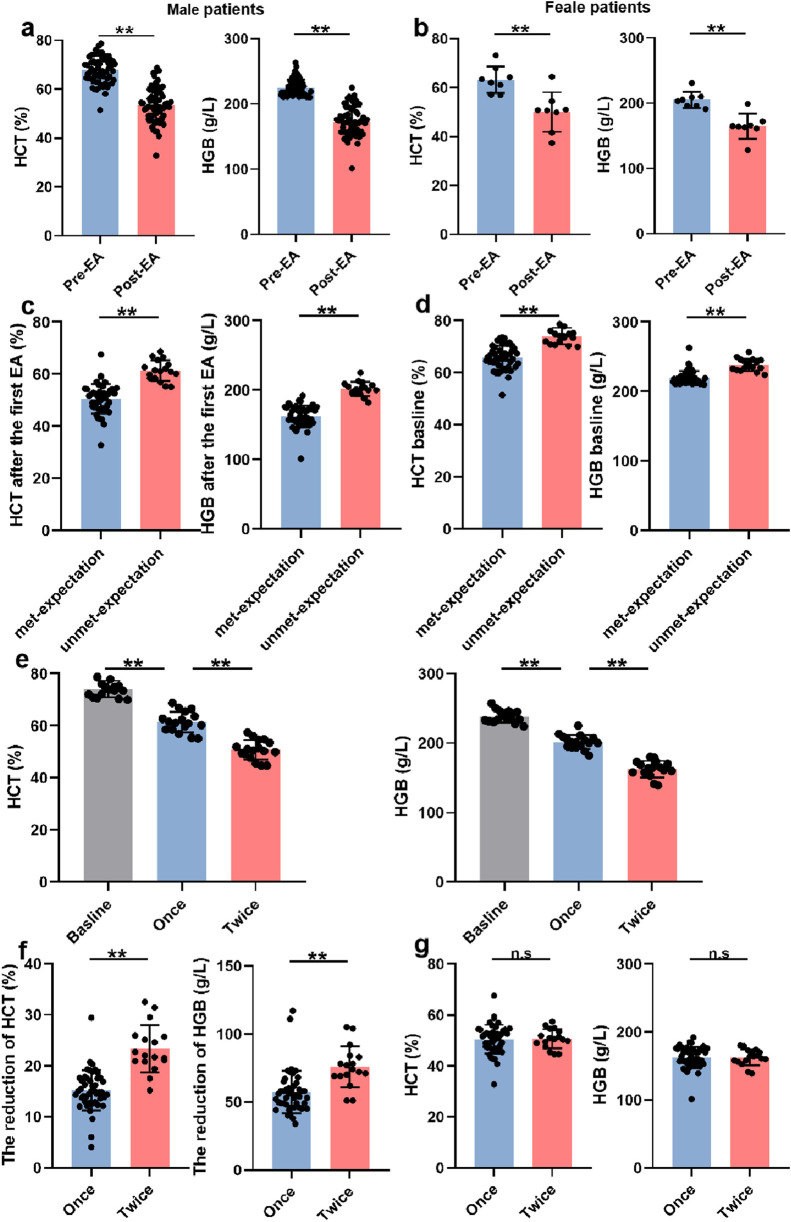
EA improved HAPC -related parameters. **(a)** The HCT and HGB levels before and after the first EA in male patients (n = 63). **(b)** The HCT and HGB levels before and after the first EA in female patients (n = 8). **(c)** The HCT and HGB levels in male patients met-expectation (n = 46) or unmet-expectation (n = 17) after the first EA. **(d)** The baselines of HCT and HGB in male patients met-expectation (n = 46) or unmet-expectation (n = 17) after the first EA. **(e)** The HCT and HGB levels in patients who needed twice EA (Once and twice mean the first and second EA, n = 17). **(f)** The degree of reduction in HCT and HGB levels caused by once (n = 46) or twice (n = 17) EA. **(g)** The final HCT and HGB levels in experienced once (n = 46) or twice (n = 17) EA patients.

To evaluate the therapeutic efficacy of EA in SHADAS patients with HAPC, we initially analyzed HCT and HGB levels. As hypothesized, a single EA session significantly reduced HCT and HGB levels in both male and female patients (males: ΔHCT −15.23% ± 3.95%, ΔHGB −57.33 ± 15.67 g/L; females: ΔHCT −13.21% ± 3.27%, ΔHGB −40.38 ± 9.91 g/L; P < 0.001; Figures 2a,b). However, 18 of 71 patients (25.4%) required a second EA due to the reduction of HCT and HGB did not meet expectations post-initial treatment (HCT 50.54% ± 5.65% vs 61.40% ± 3.97%; HGB 162.35 ± 15.62 g/L vs 201.53 ± 10.07 g/L; P < 0.001; Figure 2c). Notably, baseline HCT and HGB levels were higher in patients who did not meet treatment expectations after the initial session (males: HCT 65.76% ± 5.53% vs 74.12% ± 3.17%; HGB 219.67 ± 9.98 g/L vs 238.47 ± 8.76 g/L; P < 0.001; Figure 2d), indicating that HAPC severity may influence EA efficacy. Among theses patients, 17 were males and one was female (P > 0.05), indicating that the efficacy of EA did not correlate with sex (Table 2). Moreover, in the analysis of their ancestral origin, it was not found that had an effect on the frequency of EA (Table 3).

**TABLE 2:** Relationship between sex and the frequency of EA.

Frequency of EA	Male (n, years)	Female (n, years)
1	46 (39.56 ± 14.80)	7 (62.00 ± 9.36)
2	17 (40.06 ± 12.67)	1 (23)

The efficacy of EA, did not correlate with patients sex.

**TABLE 3:** Relationship between ancestral home and the frequency of EA.

Frequency of EA	Naives (n)	Migrators (n)
1	9	44
2	5	13

The efficacy of EA, did not correlate with patients ancestral home.

Although twice EA achieved greater cumulative reductions in HCT and HGB than a single EA (ΔHCT −15.23% ± 3.95% vs. −23.38% ± 4.64%; ΔHGB −57.32 ± g15.67 g/L vs. −75.88 ± 15.06 g/L; P < 0.001; Figures 2e,f), final post-treatment values did not differ significantly between groups (HCT 50.54% ± 5.65% vs 50.74% ± 3.77%; HGB 162.35 ± 15.62 g/L vs 162.59 ± 11.53 g/L; P > 0.05; Figure 2g). Collectively, these findings suggest that while erythrocytosis severity modulates EA responsiveness, escalating treatment frequency could overcome this limitation. EA demonstrated consistent therapeutic efficacy across all HAPC severity strata, with universal symptomatic improvement in HADAS manifestations.

### EA relieved HADAS symptoms

To further assess EA’s impact on SHADAS, we compared symptom severity before and 1 week post-EA using a standardized scoring system (scores ≤1 defined as complete remission). Consistent with hematologic improvements, EA significantly alleviated all HADAS symptoms, including marked reductions in headache (pre-EA 88.73% vs post-EA 16.90%; P < 0.001) and insomnia (pre-EA 85.92% vs post-EA 30.99%; P < 0.001, Table 4). Importantly, no symptom recurrence was observed during follow-up. These findings highlight that EA-mediated direct red blood cell removal not only normalizes hyperviscosity but also rapidly resolves hypoxia-reoxygenation injury-driven symptoms. The marked symptom resolution observed across the cohort establishes EA as a powerful therapeutic modality, effectively normalizing hematologic derangements and directly improving HADAS symptoms in SHADAS patients accompanied by HAPC.

**TABLE 4:** Improvement of HADAS symptoms by EA.

Symptoms	Pro-EA (n, %)	1 week Post-EA (n, %)	P
Fatigue	52 (73.24%)	28 (39.44%)	<0.01
Dizziness, headache	63 (88.73%)	12 (16.90%)	<0.01
Palpitations	49 (69.01%)	23 (32.39%)	<0.01
Insomnia or Lethargy	61 (85.92%)	22 (30.99%)	<0.01
Loss of appetite	50 (70.42%)	25 (35.21%)	<0.01

EA, treatment rapidly attenuated all the symptoms of HADAS, especially headache and insomnia.

## Discussion

This study demonstrates a significant clinical association between HADAS and HAPC manifestations. Notably, our clinical data results reveal that EA effectively mitigates HADAS symptoms via accelerated erythrocyte clearance, potentially linked to reduced red blood cell concentration and blood viscosity, thereby alleviating hyperviscosity-induced organ damage (Tremblay et al., 2019; Ramo et al., 2012). However, the specific mechanisms require further validation. Collectively, these findings suggest HAPC contributes to HADAS pathogenesis, with erythrocytosis serving as a principal driver. Thus, targeting HAPC pathophysiology holds substantial preventive value for HADAS, while EA emerges as a promising clinical intervention strategy for acute HADAS management.

As previously noted, prolonged exposure to high altitude hypoxia induces erythrocytosis and increased blood viscosity, manifesting as dizziness, headache, and dyspnea-hallmarks of HAPC (Villafuerte et al., 2022). Multiple mechanisms are involved in the occurrence of HAPC, with hypoxia induced factor (HIF) plays an important role (Li and He, 2019; Li et al., 2024). Studies indicate the HIF-erythropoietin (EPO) pathway may be the primary driver (Li et al., 2024; Su et al., 2015). Studies indicate the HIF-erythropoietin (EPO) pathway as a primary driver: HIF stimulates renal and hepatic EPO secretion, which activates the janus kinase 2 (JAK2)-signal transducer and activator of transcription 5 (STAT5) pathway to promote erythrocyte proliferation via upregulated membrane protein, cytoskeleton, and hemoglobin expression (Cokic et al., 2012; Held et al., 2020; Lai et al., 2005). Chronic hypoxia also upregulates activator protein-1 (AP-1) and nuclear factor-kappa B (NF-κB), inducing vascular endothelial growth factor (VEGF), endothelin-1 (ET-1), and glucose transporter protein (GLUT) to modulate the hematopoietic microenvironment (Qu et al., 2021). Nevertheless, the mechanism of erythrocytosis caused by high altitude environment remains to be further uncovered.

HADAS refers to a series of reactions after returning to low altitude regions (He et al., 2019; Paul et al., 2023). Especially for HAPC patients, the incidence of HADAS is up to 3/4. These reactions include fatigue, lethargy, insomnia, memory loss, headache, throat discomfort, cough, chest tightness, palpitation, increased or decreased appetite, constipation, diarrhea, abdominal distension, abdominal pain, back and joint pain. Almost covers the nervous, digestive and circulation systems (He et al., 2016). In severe cases, symptoms may persist for years, forcing individuals to return to high altitude regions. The underlying mechanisms of HADAS may be related to excess oxygen and blood viscosity. Usually, when the people living at high altitude returned to the low altitude regions, oxygen content in the air increased rapidly. At this time, body no longer need additional red blood cells to transport oxygen, so the production of red blood cells gradually decreased accompanied by plasma content increase. However, the body cannot reach a state of equilibrium quickly as expected, and hypoxia-oxygen-enriched injury appeared (Granger and Kvietys, 2015). In oxygen-rich environment, the production of reactive oxygen species (ROS) is increased, and cause damage to various organs such as Heart, kidney, liver and brain (Cadenas, 2018; Jiang et al., 2021). These mechanisms partly explain HADAS prevalence in HAPC patients, as elevated erythrocyte and blood viscosity exacerbates tissue injury.

The expanding research focus on HADAS has facilitated the establishment of systematic prevention and treatment protocols. Current clinical guidelines prioritize graded altitude descent as the effective intervention, which mitigates reoxygenation stress by progressively normalizing blood oxygen saturation levels, thereby avoiding complications from abrupt atmospheric adaptation (Wang et al., 2024). Hyperbaric oxygen therapy represents another validated therapeutic modality, delivering supraphysiological oxygen concentrations to rapidly correct tissue hypoxia, elevate arterial oxygen tension, and ameliorate hypoxia-induced neurological impairments such as central fatigue and neuronal injury (Jin and Chen, 2000). Nevertheless, these conventional therapeutic modalities face significant implementation barriers in complex emergency scenarios, particularly in natural hazard requiring rapid resolution for affected populations. Our results demonstrate that therapeutic EA directly addresses HADAS pathophysiology through targeted erythrocyte depletion, achieving rapid symptomatic relief. However, the impact of EA on molecules closely associated with HADAS occurrence, such as IL-17A, still remains to be further studied and explored (He et al., 2019; He et al., 2016). This mechanistically driven approach positions EA as a frontline intervention for SHADAS patients with comorbid HAPC, particularly in emergency management scenarios demanding immediate therapeutic efficacy. It enables rapid relief for severe cases unable to return to high-altitude environments.

This study also has some limitations. Due to the single-center data, the sample size of patients is small, and the gender composition is dominated by males, and the age of females is higher than that of males, so the efficacy of EA in different genders remains to be further verified. We hope that more institutions will join in the future to investigate the occurrence mechanism of HADAS and provide clinical evidence for EA in the treatment of SHADAS.

## Conclusion

Our study demonstrates that EA constitutes a clinically viable therapeutic approach for SHADAS patients accompanied by HAPC, which could effectively reducing pathological erythrocytosis and concomitantly ameliorating systemic decompensation symptoms.

## Data Availability

The original contributions presented in the study are included in the article/supplementary material, further inquiries can be directed to the corresponding authors.

## Abbreviations

AP-1, Activator protein-1; EA, Erythrocyte apheresis; ET-1, Endothelin-1; EPO, Erythropoietin; GTP, Glucose transporter protein; HADAS, High-altitude deacclimatization syndrome; HAPC, High altitude erythrocytosis; HCT, hematocrit; HGB, Hemoglobin; HIF, Hypoxia induced factor; JAK2, Janus kinase 2; NF-kB, Nuclear factor-kappa B; ROS, Reactive oxygen species; VEGF, Endothelial growth factor.
